# Supplementary material for: A novel use of HIV surveillance and court data to understand and improve care among a population of people with HIV experiencing criminal charges in North Carolina 2017–2020
Source: medRxiv. 2024 Apr 15:2024.04.14.24305790. Preprint. [Version 1] doi: 10.1101/2024.04.14.24305790 (PMC11065039; doi:10.1101/2024.04.14.24305790)
Supplement: Supplement 1 [file NIHPP2024.04.14.24305790v1-supplement-1.pdf]

| Supplementary Table 1. Department of public health versus Court record coding of race/ethnicity | | | | | | | | | |
|---|---|---|---|---|---|---|---|---|---|
| | | Department of Public Health Race/ethnicity coding | | | | | | | |
| Court Race/ethnicity coding | | Black, non-Hispanic | Black, Hispanic | White, Hispanic | White, non-Hispanic | Asian | American Indian/Alaska native | Other or unknown | **Row totals** |
| | Black | 7226 | 53 | 10 | 47 | 6 | 11 | 0 | 7353 |
| | White | 41 | 2 | 58 | 1742 | 3 | 14 | 0 | 1860 |
| | Hispanic | 7 | 2 | 109 | 11 | 3 | 0 | 1 | 133 |
| | Other or Unknown | 70 | 6 | 15 | 52 | 21 | 0 | 4 | 168 |
| | **Column totals** | 7344 | 63 | 192 | 1852 | 33 | 25 | 5 | |

medRxiv preprint doi: https://doi.org/10.1101/2024.04.14.24305790; this version posted April 15, 2024. The copyright holder for this preprint (which was not certified by peer review) is the author/funder, who has granted medRxiv a license to display the preprint in perpetuity.
It is made available under a CC-BY 4.0 International license .

**Supplementary Table 2. Baseline characteristics of people with HIV without full follow-up periods (n=2,847)**

| | n (col%) or median (1st-3rd quartiles) |
|---|---|
| **Sex** | |
| Male | 2,228 (78.3) |
| Female | 619 (21.7) |
| **Race** | |
| White | 568 (20.0) |
| Black | 2,204 (77.4) |
| Hispanic | 26 (0.9) |
| Other or Unknown | 49 (1.7) |
| **Age (years)** | 38 (29-50) |
| **Charge days** | 78 (1-257) |
| **HIV transmission group** | |
| MSM | 1,305 (45.8) |
| Unknown | 771 (27.1) |
| Heterosexual contact | 410 (14.4) |
| IDU | 203 (7.1) |
| MSM & IDU | 138 (4.8) |
| Other | 20 (0.7) |

Abbreviations: MSM, men who have sex with men; IDU, injection drug use; IQR interquartile range

| Supplementary Table 3. Association[1] between criminal charge period and duration of unresolved charges NC 2017-2020: Results of multivariable log-binomial model (n=6,427)[2] Analysis including data from all NC counties. | | | |
|---|---|---|---|
| Duration of unresolved charges in days | n (%) | % with viral suppression pre/post charge period | RR of viral suppression in post-charge period compared to pre-charge period |
| 0-99 | 4,237 (66) | 69.8/71.8 | 1.03 (1.01-1.04) |
| 100-199 | 984 (15) | 72.4/74.3 | 1.03 (0.90-1.06) |
| 200-299 | 592 (9) | 68.6/72.6 | 1.06 (1.01-1.10) |
| 300+ | 604 (10) | 64.4/68.1 | 1.06 (1.01-1.12) |

[1] Multivariable log-binomial estimates
[2] Only individuals who contributed person-time to both pre- and post-charge periods and who had a single period of criminal charges were included in model

medRxiv preprint doi: https://doi.org/10.1101/2024.04.14.24305790; this version posted April 15, 2024. The copyright holder for this preprint (which was not certified by peer review) is the author/funder, who has granted medRxiv a license to display the preprint in perpetuity.
It is made available under a CC-BY 4.0 International license .

| Supplementary Table 4. Baseline characteristics of people with and without HIV who charged in criminal court in NC between 2017-2020. Data limited to 26 counties with jail incarceration data available | | | | | | |
|---|---|---|---|---|---|---|
| | Jail Incarcerated and Unincarcerated | | Jail Unincarcerated Only | | Jail Incarcerated Only | |
| **Group** | Non-HIV (n=155,745) | HIV (n=1,304) | Non-HIV (n=48,846) | HIV (n=494) | Non-HIV (n=105,597) | HIV (n=989) |
| | n (col%) or median (1st-3rd quartiles) | | | | | |
| **Sex** | | | | | | |
| Male | 118,267 (75.9) | 1,087 (83.4) | 37,781 (77.3) | 404 (81.8) | 79,399 (75.2) | 832 (84.1) |
| Female | 37,478 (24.1) | 217 (16.6) | 11,065 (22.7) | 90 (18.2) | 26,198 (24.8) | 157 (15.9) |
| **Race** | | | | | | |
| White | 72,127 (46.7) | 267 (20.5) | 20,787 (42.6) | 92 (18.6) | 51340 (48.4) | 214 (21.6) |
| Black | 72,805 (47.1) | 1,005 (77.1) | 25,556 (52.3) | 391 (79.1) | 47,997 (45.0) | 748 (75.6) |
| Hispanic | 5,582 (3.6) | 12 (0.9) | 1,415 (2.9) | 3 (0.6) | 4,177 (3.9) | 10 (1.0) |
| Other or Unknown | 3,034 (2.0) | 20 (1.5) | 1,088 (2.2) | 8 (1.6) | 2,158 (2.0) | 17 (1.7) |
| **Age (years)** | 31 (25-40) | 37 (28-49) | 31 (25-40) | 38 (28-49) | 31 (24-40) | 37 (28-48) |
| **Total number of charge periods** | | | | | | |
| 1 | 129,870 (84.1) | 1,301 (99.8) | 35,352 (72.4) | 492 (99.6) | 94,518 (89.5) | 986 (99.7) |
| 2 | 21,457 (13.9) | 3 (0.2) | 11,509 (23.6) | 2 (0.4) | 9,948 (9.4) | 3 (0.3) |
| 3 or greater | 3,114 (2.0) | 0 | 1,985 (4.1) | 0 | 1,129 (1.1) | 0 |
| **Charge days** | 139 (56-270) | 121 (37-277) | 91 (25-40) | 65 (2-182) | 165 (73-301) | 161 (64-309) |
| **HIV transmission group** | | | | | | |
| MSM | | 506 (38.8) | | 183 (37.0) | | 387 (39.1) |
| Unknown | | 414 (31.7) | | 151 (30.6) | | 319 (32.3) |
| Heterosexual contact | | 175 (13.4) | | 74 (15.0) | | 125 (12.6) |
| IDU | | 125 (9.6) | | 60 (12.1) | | 90 (9.1) |
| MSM & IDU | | 78 (6.0) | | 24 (4.9) | | 63 (6.4) |
| Other | | 6 (0.5) | | 2 (0.4) | | 5 (0.5) |

Abbreviations: NC, North Carolina; MSM, men who have sex with men; IDU, injection drug use; IQR interquartile range.

**Supplementary Table 5. Viral suppression outcome[1] among people with HIV (PWH) in NC with a single criminal charge between 2017-2020: results of multivariable log-binomial model limited to NC counties with available jail incarceration data[2].**

| | 26 counties where jail incarceration data available (n=857) | Unincarcerated during charge period (n=246) | Incarcerated during charge period (n=611) |
|---|---|---|---|
| | **Adjusted RR (95% CI)[3]** | **Adjusted RR (95% CI)[3]** | **Adjusted RR (95% CI)[3]** |
| **Criminal Charge Group** | | | |
| 12 months pre charge | | Reference | |
| 12 months post charge | 0.97 (0.90-1.04) | 0.98 (0.89-1.08) | 0.98 (0.89-1.07) |
| **Age (years)** | | | |
| 18-29 | | Reference | |
| 30-39 | 1.11 (0.96-1.28) | 0.96 (0.80-1.14) | 1.19 (0.98-1.44) |
| 40-49 | 1.28 (1.10-1.49) | 1.01 (0.81-1.26) | 1.41 (1.16-1.71) |
| 50-59 | 1.49 (1.28-1.74) | 1.15 (0.94-1.42) | 1.63 (1.33-2.00) |
| 60-69 | 1.26 (0.93-1.71) | 0.91 (0.57-1.44) | 1.41 (0.96-2.07) |
| 70 and older | NA | NA | NA |
| **Race/Ethnicity** | | | |
| White | | Reference | |
| Black | 0.83 (0.73-0.93) | 0.85 (0.70-1.03) | 0.82 (0.71-0.96) |
| Hispanic | 1.59 (1.35-1.89) | 1.59 (1.23-2.05) | 1.52 (1.15-2.01) |
| Other | 0.93 (0.60-1.44) | 0.81 (0.39-1.69) | 1.60 (0.84-1.59) |
| **Sex** | | | |
| Male | | Reference | |
| Female | 0.85 (0.72-1.01) | 0.92 (0.72-1.18) | 0.79 (0.63-0.99) |
| **HIV Transmission Category** | | | |
| MSM | | Reference | |
| Heterosexual | 1.00 (0.85-1.88) | 1.66 (0.89-1.49) | 0.96 (0.77-1.17) |
| IDU | 0.81 (0.67-0.98) | 0.93 (0.72-2.00) | 0.78 (0.60-1.00) |
| MSM and IDU | 1.02 (0.82-1.26) | 1.12 (0.84-1.50) | 0.98 (0.77-1.25) |
| Other | 0.99 (0.77-1.00) | 0.93 (0.80-1.09) | 1.24 (1.06-1.45) |

[1] Multivariable log-binomial model estimates, adjusting for all listed variables.[2] Only individuals who contributed person-time to both pre- and post-charge periods and who had a single period of criminal charges were included in

model. [3]Adjusted RRs are adjusted for all the variables in the table. Abbreviations: RR, risk ratio; CI, confidence interval; HIV, human immunodeficiency virus; MSM, men who have sex with men; IDU, injection drug use. Definitions: Viral Suppression: <200 copies of HIV RNA per milliliter of blood
